# Editorial

**Published:** 1916-09

**Authors:** 


### EDITORIAL

The Atlanta Journal-Record of Medicine has its Business Office at 23 West Alabama Street where all business communications should be sent.

Remittances are payable to The Journal-Record of Medicine.

Reprints of Original Articles will be furnished at cost price. Requests for them had best be made on the Manuscript.

Upon request, we will present, postpaid, to each contributor of an original article, twenty copies of The Journal-Record of Medicine containing such article.

---

### ANNUAL MEETING OF THE SOUTHERN MEDICAL ASSOCIATION

The Association will have its annual meeting at Atlanta on November 13 to 16 inclusive.

This statement should attract the attention of everyone interested in medical meetings and in the progress of medical science and sanitation in the South. The ideal of the organization is a ''Greater South through a healthier South'' and there should be no better one. That it is lived up to by the members has been proved by the steady growth of the Association and by the work it has done at its meetings, by its various Committees and through the dissemination of its information by its authorized Journal. The Association needs no praise from us but we have some pledges of our own to make and we take this occasion to say to every physician contemplating attendance upon the meeting that Atlanta has plans and ideals of her own and that they comprise making every visitor comfortable and happy.

Not alone the doctors, but the business and other professional men are interested. We all feel a pride in having the Association meet here and we realize that out reputation as a convention city is at stake.

A prominent feature of the meeting will be the clinics held every morning from 8 to 10 at the various hospitals by the best known men of other cities. Every possible facility will be afforded them for doing whatever work they are best equipped to demonstrate and of course similar arrangements will be made for visitors to see them. We hope to have exhaustive clincs in all the branches thus presenting in the most practical way the latest things experience has taught. On Friday and Saturday following the meetings clinics will be held all day.

The Section Programs have already been arranged sufficiently to insure a very interesting group of sessions. Moreover, all the sessions and section meetings will be held under one roof so that there can be no inconvenience in getting about. The Auditorium is large enough for all and at the same time it will give enough privacy to avoid confusion.

On Monday the Railway Surgeons, Public Health Section and teh Conference on Medical Education will be in session. Monday evening there will be a public meeting with addresses by prominent health workers. Tuesday morning, addresses of welcome and the formal opening exercises. Tuesday night, a reception at the Capital City Club. Wednesday, a ''Georgia Barbecue'' at the Druid Hills Country Club. This Club has fine golf link and those who wish to play will have the privilege that afternoon. There are two other fine links, the East Lake and the Brookhaven, that will be open to visitors during the Meeting. There will be a golf tournament on Friday. However, entertainments are so arranged as not to interfere with the scientific sessions.

We can do no more than hint at the fine things the whole program offers, but we can say that all of Atlanta's goodwill is given to this meeting and that it will be shown in the hospitality and welcome we are eager to extend to our visitors. If this means anything to our readers, we hope that it will induce them to attend. If any are not yet members of the Southern Medical Association and are eligible, they can secure membership by applying to Dr. Seal Harris at Birmingham or to Dr. Montague Boyd at Atlanta.

Special entertainment has been planned for the ladies and a Woman's Health Conference will be in session. The complete program will be sent out as soon as finished.

The Chairman of the Committee on Hotels.

Dr. G. Pope Huguley, Atlanta National Building., Atlanta.

The Chairman of the Committee on Transportation is Dr. L. L. Clark, Peters Building, Atlanta. All the railroads east of the Mississippi have made special rates and it is expected that these in the west will do the same.

